# Ventricular Tachyarrhythmia in a Newborn Baby

**DOI:** 10.1093/omcr/omae103

**Published:** 2024-09-07

**Authors:** Alexander Paul Henning Maclennan, Emily Sitner-Medvedovsky, Daryelle Varon, Abhinav Parikh, Ankur Shah, Manoj Chhabra

**Affiliations:** Department of Pediatrics, NewYork-Presbyterian Brooklyn Methodist Hospital, 506 6^th^ Street, Brooklyn, NY 11215, United States; Department of Pediatrics, NewYork-Presbyterian Brooklyn Methodist Hospital, 506 6^th^ Street, Brooklyn, NY 11215, United States; Department of Pediatrics, NewYork-Presbyterian Brooklyn Methodist Hospital, 506 6^th^ Street, Brooklyn, NY 11215, United States; Department of Pediatrics, NewYork-Presbyterian Brooklyn Methodist Hospital, 506 6^th^ Street, Brooklyn, NY 11215, United States; Department of Pediatrics, NewYork-Presbyterian Brooklyn Methodist Hospital, 506 6^th^ Street, Brooklyn, NY 11215, United States; Department of Pediatrics, NewYork-Presbyterian Brooklyn Methodist Hospital, 506 6^th^ Street, Brooklyn, NY 11215, United States

**Keywords:** cardiology, cardiovascular systems, and neonatology

## Abstract

Supraventricular tachycardia (SVT) is a narrow QRS complex tachyarrhythmia with a heart rate above 220 beats per minute in infants and children. Ventricular tachycardia can be due to electrolyte abnormalities, cardiomyopathies, congenital heart disease, myocarditis or drug toxicity. Incidence has been estimated to be 1 in 250 to 1 in 1000 with spontaneous resolution in infants by one year of life. We present a full-term neonate who started experiencing a tachyarrhythmia on day zero of life until she reverted to sinus rhythm on day nine of life. The rhythm was most likely a ventricular tachyarrhythmia rather than supraventricular tachycardia due to its unresponsiveness to adenosine, wide QRS complexes, and lack of association with hemodynamic instability. This is a unique case presentation of a ventricular tachyarrhythmia for its diagnostic and therapeutic challenges, it’s idiopathic nature and lack of association with any cardiac compromise, congenital heart disease or electrolyte imbalance.

## Case

**Figure 1:**
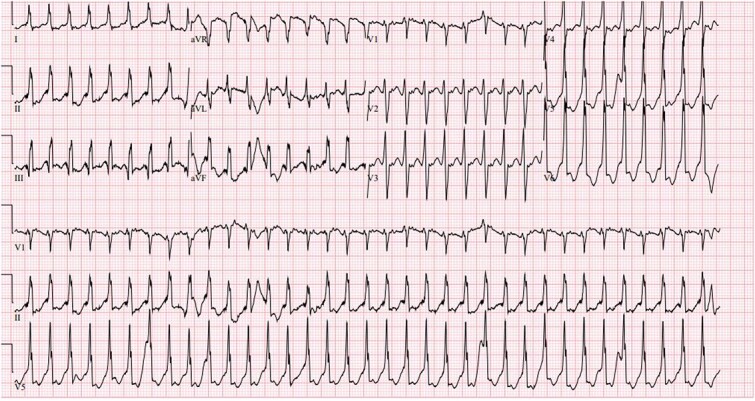
Electrocardiogram on DOL 0 (before Adenosine); Wide complex QRS suggestive of possible delta waves.

**Figure 2:**
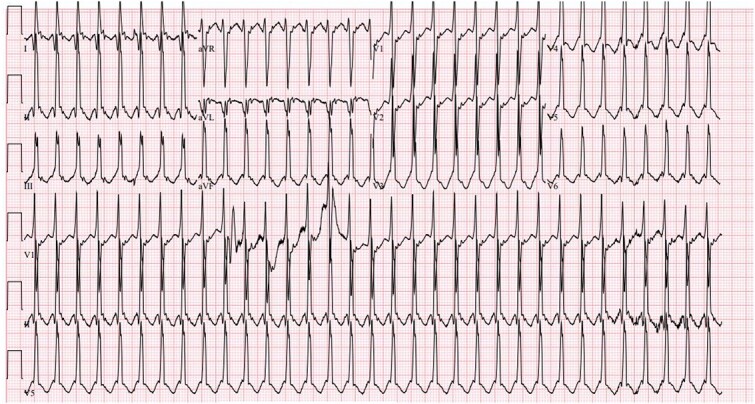
Electrocardiogram during Adenosine administration.

**Figure 3:**
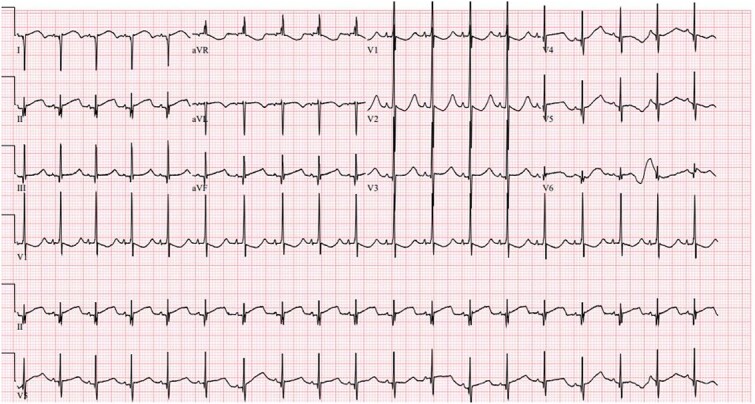
Electrocardiogram after cardioversion.

A 37 week female born to a gravida 3 para 1 mother with no medical history and negative maternal labs presented with heart rate consistently above 200 beats per minute at birth. APGAR scores were 8 at 1 min and 9 at 5 min, and routine resuscitation was done per NRP guidelines. Physical exam was unremarkable except for a 4 centimeter by 4 centimeter blanching hemangioma on the back. The newborn was brought to the neonatal intensive care unit where EKG showed a heart rate of 235 beats per minute (BPM), broad QRS tachycardia with P-waves coming after the QRS, and possible delta waves (Fig. 1). Both ice and the gag reflex did not break the SVT. It was also resistant to multiple doses of intravenous (IV) adenosine at escalating doses, so the baby was treated with synchronized cardioverted which converted her back to normal sinus rhythm. The patient was then placed on IV procainamide drip and oral propranolol. She remained free of tachyarrhythmias for 24 h, so the procainamide was discontinued. As the EKG demonstrated a wide-complex tachycardia, the first differential was ventricular tachycardia versus an SVT with aberrant conduction. Since SVT is more common in neonates, the initial treatment approach assumed that the tachyarrhythmia was SVT. Vagal maneuvers were attempted followed by adenosine. Administration of Adenosine (Fig. 2) showed 1:1 conduction with no evidence of atrio-ventricular dissociation; this finding is consistent with SVT. However, increasing doses of Adenosine failed to convert to sinus rhythm so cardioversion was utilized (Fig. 3).

On day 2 of life, the baby had multiple recurrences of wide-complex tachycardia with P-waves coming after the QRS at a rate of approximately 200 BPM, similar in appearance on EKG to the first episode. These episodes were likewise resistant to escalating doses of IV adenosine, so the baby was treated with synchronized cardioversion each recurrence for a total of 2 additional times. The dosage of propranolol was increased and the baby was placed back on IV procainamide initially. After review by an electrophysiologist, the decision was made to substitute IV procainamide with IV amiodarone. On day 3 of life, EKG again showed wide QRS tachycardia at a rate of 170 BPM, which then resolved on its own on day 9 of life. An echocardiogram done during the patient’s admission showed no evidence of cardiomegaly or intracardiac masses. Given neonates unrepsonsiveness to adenosine the working diagnosis for the wide-complex tachycardia was ventricular tachycardia (which is rare in this age group) rather than supraventricular tachycardia. The baby was discharged home on both oral amiodarone and propranolol. After multiple follow up appointments, these medications were both discontinued, and the baby continues to do well off of medications. She continues to be monitored closely by cardiology.

## Discussion

The normal fetal heart rate can range from 120–160 bpm, with rates less than 100 bpm considered as bradycardia, and rates greater than 180 bpm are tachycardia. They will be noted on maternal examination and more often than not the mother will be asymptomatic and will not report any changes in fetal activity [1]. This proved to be true in our case as the mother presented to her maternal fetal specialist for routine examination where it was discovered that the fetus had a heart rate in the 200 s without any changes in activity. Fetal arrhythmias may occur at any point in fetal life, but most commonly occur after 22 weeks gestation. It is considered normal in the first week of life that the neonatal heart rate can range from 90–160 bpm with a PR interval 0.08–0.15 s, and QRS complex of 0.03–0.08 s [2].

Arrhythmias in the neonatal period may occur in neonates with or without structural heart disease. Literature review demonstrates that the incidence is 1%–5% of all neonates. Neonates who present with wide QRS complex tachycardia on ECG should be assumed to be in ventricular tachycardia (VT) though ventricular tachycardia is rare. Supraventricular tachycardia (SVT) with aberrancy, SVT in an antidromic tachycardia or SVT with the presence of an underlying bundle branch block or ventricular pre-excitation can also be considered. VT is defined as three or more premature ventricular contractions (PVC) in a row at a heart rate greater than 120 bpm, with a broad QRS complex with AV dissociation [3]. Sometimes a sinus beat will pass through the AV node and “capture” the bulk of the ventricle, which leads to an isolated narrow complex beat called a “capture” beat which is pathognomonic of ventricular tachycardia [4]. Causes of ventricular tachycardia include cardiac tumors, congenital heart disease, myocarditis, long QT syndrome and electrolyte abnormalities, none of which our patient was found to have.

Supraventricular tachycardia is the most common arrhythmia in infants and children, where the heart rate can be greater than 220 bpm. ECG findings are consistent with a narrow complex tachycardia, without P waves or with retrograde P waves. The most common type of SVT is AV reentrant tachycardia, where there is an accessory bypass pathway and conduction through this pathway does happen more rapidly than through the normal conduction pathway which creates a pattern of cyclic reentry independent of the SA node [2]. Wolf-Parkinson White syndrome (WPW) is the most common explanation for SVT in the pediatric population, as it is a form of AV reentrant tachycardia. Initially, it was thought this was what our patient had due to her wide complex tachycardia with aberrant conduction due to persistent 1:1 conduction with no transient ventricular-atrial (VA) dissociation noted during administration of Adenosine. Additionally, a baseline EKG shortly after birth showed a shortened PR interval in lead V2, suggesting ventricular pre-excitation at baseline (WPW syndrome). Neonates and infants who present with SVT the heart rate can be between 220–280 bpm. Our patient’s heart rate varied from 190–220 immediately after delivery, reaching as high as 220 s–230 s during hospital stay prior to spontaneous conversion to sinus rhythm, after which it ranged from 120 s–170 s bpm.

This case was challenging because EKG findings and the clinical course did not clearly indicate the etiology of the tachyarrhythmia. The lack of AV disassociation after administration of adenosine is consistent with SVT. However, the main differentiation between SVT and VT in our patient was that she was unresponsive to multiple rounds of Adenosine. Literature review proves that Adenosine is both diagnostic and therapeutic for SVT. Adenosine causes cessation of an AV reciprocating tachycardia which uses the AV node block. It is appreciated for its short half life (<1.5 s) [1]. Adenosine is not useful in treating VT. On DOL 0, our patient was given six doses of Adenosine before the decision was made to cardiovert. On DOL 4, Adenosine was given four times prior to cardioversion with successful return to sinus rhythm. Our patient continued to have wide complex tachycardia with rates of at least 190 bpm due to failure to convert with Adenosine. Due to failure to convert her tachyarrhythmia with Adenosine and repeated need for conversion, our patient’s tachyarrhythmia was likely ventricular in origin. Subsequent ECG’s after initial cardioversion on DOL 0 did not demonstrate pre-excitation. Though our patient did not have capture beats that are described in the literature to be pathognomonic of VT, her failure to respond to Adenosine is likely due to the fact that her tachycardia was not SVT. Infants may present with nonspecific complaints when there is an underlying cardiac abnormality, such as increased fussiness, poor feeding, sweating with feeds or lethargy. Our patient remained well-appearing and did not present with or develop other cardiac symptoms other than tachycardia during her hospital stay. Further evaluation of SVT includes complete blood count, electrolyte levels, blood gas, and thyroid function tests. All these laboratory evaluations for our patient were within normal limits, and echocardiograms repeatedly demonstrated a structurally normal heart.

Our patient has continued to follow up with an electrophysiologist specialist, the most recent visit at three months of life. A 15 lead ECG done during the visit demonstrated a heart rate of 15 bpm with baseline artifact. A normal axis was noted with no evidence of ventricular pre-excitation. There was no suggestion of atrial or ventricular enlargement and hypertrophy. The QT interval was about 455 ms. Prognosis for neonates and infants with ventricular arrhythmias are generally optimistic. Antiarrhythmic medication maintains the heart rate within appropriate limits, and procedures such as radiofrequency catheter ablation can be curative, with success rates more than 90% immediately after the procedure, and around 71%–77% three years afterward [5]. Our patient was discharged from the hospital on a beta blocker, Propranolol which was discontinued at three months of life and a class III antiarrhythmic drug, Amiodarone, which was discontinued at two months of life. She has remained without tachyarrhythmias with a structurally normal heart and no identifiable cause for her ventricular tachycardia.

## Conclusion

Ventricular tachyarrhythmias are often associated with hemodynamic instability, and can be due to cardiomyopathies, congenital heart disease, prolonged QT syndrome and electrolyte abnormalities. Our patient was not found to have any aforementioned defect and did not experience any hemodynamic compromise during tachycardic episodes. Serial echocardiograms monitored cardiac function which did not note any abnormalities. The lack of response to Adenosine, and positive response to Amiodarone, Procainamide and Propranolol supports the diagnosis of ventricular tachyarrhythmia which is a rare finding in infants. Retrospectively, we can comment that as the infant was hemodynamically stable and did not respond immediately to anti-arrhytmics (continued to have an accelerated ventricular rhythm even after initation of Procainamide) less aggressive treatment may been warranted.
